# A Gold Medal

**Published:** 1870-12

**Authors:** 


*A Gold Medal* is offered by the "Odontological Society of London," for the best essay on the minute structure of the teeth. Essays are to be sent under a motto, accompanied by a sealed envelope containing the author's name and address under the same motto, to the President of the Society, 31 Soho Square, London, before the 31st of December 1871. We express the hope that this medal may come into the possession of an American.

